# Fibrosis-5 predicts end-stage renal disease in patients with microscopic polyangiitis and granulomatosis with polyangiitis without substantial liver diseases

**DOI:** 10.1007/s10238-021-00691-2

**Published:** 2021-02-20

**Authors:** Hyeok Chan Kwon, Jason Jungsik Song, Yong-Beom Park, Sang-Won Lee

**Affiliations:** 1 Department of Rheumatology, Dankook University Hospital, Dankook University College of Medicine, Cheonan, Republic of Korea; 2 Division of Rheumatology, Department of Internal Medicine, Yonsei University College of Medicine, 50-1 Yonsei-ro, Seodaemun-gu, Seoul, Republic of Korea; 3 Institute for Immunology and Immunological Diseases, Yonsei University College of Medicine, Seoul, Republic of Korea

**Keywords:** Microscopic polyangiitis, Granulomatosis with polyangiitis, FIB-5, End-stage renal disease, Prediction

## Abstract

We previously reported that fibrosis-4 (FIB-4) was associated with poor outcomes of microscopic polyangiitis (MPA) and granuloma with polyangiitis (GPA). We also investigated the potential of FIB-5, a novel index, in predicting all-cause mortality and end-stage renal disease (ESRD) during follow-up in patients with MPA and GPA without substantial liver diseases. Clinical and laboratory data at diagnosis were collected by reviewing the medical records of 180 patients with MPA and GPA. FIB-5 was obtained by a following equation: FIB-5 = (serum albumin (g/L) × 0.3 + platelet count (10^9^/L) × 0.05) − (alkaline phosphatase (IU/L) × 0.014 + aspartate aminotransferase/alanine aminotransferase ratio × 6 + 14). The median age of the patients at diagnosis was 61.0 years. FIB-5 at diagnosis could not reflect the cross-sectional vasculitis activity. The cutoffs of FIB-5 for poor outcomes was set as 0.82 (the lowest tertile) and -0.42 (the lowest quartile) at diagnosis. In Kaplan–Meier survival analysis, patients with FIB-5 < 0.82 and those with FIB-5 < -0.42 exhibited lower ESRD-free survival rates than those without. However, it could not predict all-cause mortality. In multivariable Cox hazards analysis, both FFS (Hazard ratio (HR) 1.554) and FIB-5 < 0.82 (HR 2.096) as well as both FFS (HR 1.534) and FIB-5 < -0.42 (HR 2.073) at diagnosis independently predicted ESRD during follow-up. In conclusion, FIB-5 < 0.82 and FIB-5 < -0.42 at diagnosis could predict the occurrence of ESRD, but not all-cause mortality, during follow-up in patients with MPA and GPA without substantial liver diseases.

**Supplementary information:**

The online version contains supplementary material available at 10.1007/s10238-021-00691-2.

## Introduction

Antineutrophil cytoplasmic antibody (ANCA)-associated vasculitis (AAV) is a group of systemic necrotising vasculitides encroaching small-sized vessels such as capillaries, arterioles, and venules. There are three subtypes of AAV based on the clinical and histological features—microscopic polyangiitis (MPA), granulomatosis with polyangiitis (GPA), and eosinophilic granulomatosis with polyangiitis (EGPA) [1, 2]. All-cause mortality and end-stage renal disease (ESRD) are the most serious outcomes of AAV, and the incidence rates of the two eventualities reach up to 25% and 38%, respectively [3, 4]. Therefore, it is clinically important to identify the predictors of all-cause mortality at diagnosis and occurrence of ESRD during the follow-up of AAV. To date, studies have reported several risks of ESRD in AAV patients such as MPA, myeloperoxidase (MPO)-ANCA positivity, glomerular filtration rate < 50 mL/min, and hypertension [4]. In addition, several indicators to predict all-cause mortality and relapse have been suggested in patients with AAV, such as neutrophil-to-lymphocyte ratio, initial lung and cardiovascular involvements and low serum globulin levels [5, 6]. An increase in the number of predictors of the worst outcomes of AAV would be expected to enhance the prognosis of the condition.

We have previously demonstrated that evaluation of fibrosis-4 (FIB-4) at diagnosis, one of the indices for liver fibrosis, could predict all-cause mortality in patients with MPA and GPA. However, there was no direct evidence of liver involvement, and FIB-4 value ≥ 1.45 at diagnosis was an independent predictor of all-cause mortality during the follow-up of patients with MPA and GPA (hazard ratio [HR], 6.253) [7]. In our opinion, this study provided an opportunity to recognize the relationship between a liver fibrosis index and the prognosis of MPA and GPA. Recently, another index for liver fibrosis, FIB-5, was introduced. The distinctive features of FIB-5, which is different from FIB-4, are inclusion of two variables (serum albumin and alkaline phosphatase [ALP]) and lower values are associated with a higher degree of fibrosis [8]. A previous study has reported that FIB-5 reflects the extent of liver fibrosis better than FIB-4 [9].

We suggest three hypotheses supporting our assumption that FIB-5 can predict ESRD in patients with MPA and GPA. First, myofibroblast may be activated in response to microenvironmental inflammation and autoreactive immune cell alteration, and accelerate the fibrosis of major organs by producing fibrogenic mediators. Furthermore, myofibroblast also may share the interaction with not only liver and kidney-specific cells such as portal fibroblast for liver and interstitial fibroblasts for kidneys, but also general cells participating in the fibrosis including fibrocytes and stem cells, mesothelial cells, and capillary endothelial cells [10]. Second, non-alcoholic fatty liver disease (NAFLD) is known to be associated with systemic diseases such as chronic kidney diseases as either aetiologies or consequences [11, 12]. NAFLD, in which liver cirrhosis or chronic hepatitis is not established, may also affect the serum levels of AST, ALT, and alkaline phosphatase [13]. Last, FIB-5 includes serum albumin and platelet count which may generally be used as indicators of systemic inflammation [14]. Therefore, we expect FIB-5 at diagnosis can predict ESRD occurrence during follow-up in MPA and GPA patients. However, to date, no studies have investigated the predictive potential of FIB-5 in determination of the worst outcomes during the course of MPA or GPA. Hence, in this study, we investigated the potential of FIB-5 in predicting all-cause mortality and occurrence of ESRD during follow-up in patients with MPA and GPA.

## Materials and methods

### Patients

Medical records of 196 patients with MPA and GPA were reviewed. All patients fulfilled the 2007 European Medicines Agency algorithm for AAV and polyarteritis nodosa and the 2012 revised International Chapel Hill Consensus Conference Nomenclature of Vasculitides criteria. The patients were initially diagnosed with MPA and GPA at the Department of Internal Medicine, Yonsei University College of Medicine, Severance Hospital, from October 2000 to February 2019. Their medical records were well-documented to obtain clinical and laboratory data, including demographic data, Birmingham vasculitis activity score (BVAS) version 3, five-factor score (FFS), comorbidities, ANCA positivity, and the results of routine blood tests at diagnosis [10, 11]. Patients with serious medical conditions, such as chronic liver diseases (*n* = 7), coexisting malignancies (*n* = 4), and serious infections (*n* = 3), based on the 10th revised International Classification of Diseases were excluded from this study. Patients who had received immunosuppressive drugs before diagnosis based on the Korean Drug Utilization Review system (*n* = 2) were also excluded. Finally, 180 patients with MPA and GPA were included in this study. This study was approved by the Institutional Review Board of Severance Hospital (4-2017-0673), and the need for written informed consent was waived considering the retrospective nature of the study.

### Clinical and laboratory data

Demographic data, such as age at diagnosis and sex, were recorded along with the AAV subtypes, ANCA positivity, BVAS, and FFS [15, 16]. Erythrocyte sedimentation rate (ESR), platelet count, and levels of C-reactive protein (CRP), serum albumin, alkaline phosphatase (ALP), aspartate aminotransferase (AST), and alanine aminotransferase (ALT) were obtained. Comorbidities included chronic kidney disease (stages 3–5), diabetes mellitus, hypertension and dyslipidaemia. In this study, poor outcomes of AAV were defined as all-cause mortality and ESRD during follow-up. For deceased patients, the follow-up duration based on all-cause mortality was defined as the period between the initial diagnosis of AAV and the time of death. Meanwhile, for surviving patients, it was defined as the period between the date of the diagnosis of AAV and the date of the last follow-up visit. Also, for patients with ESRD, the follow-up duration based on ESRD was defined as the period from the diagnosis of AAV until the initiation of renal replacement therapy, whereas, for patients without ESRD, it was defined as the period between the date of the diagnosis of AAV and the date of the last follow-up visit. In addition, information regarding glucocorticoid and immunosuppressive drugs administered during follow-up were recorded.

### Equation for FIB-5

FIB-5 = (serum albumin (g/L) × 0.3 + platelet count (109/L) × 0.05) − (ALP (IU/L) × 0.014 + AST/ALT ratio × 6 + 14) [8].

### Obtaining the cutoff of FIB-5 for all-cause mortality and ESRD

In principle, the optimal cutoff should be extrapolated by performing the receiver operator characteristic (ROC) curve analysis and one value having the maximized sum of sensitivity and specificity is selected. Nevertheless, when the statistically significant cutoff was not obtained, the lowest tertile or the lowest quartile of FIB-5 may be clinically used instead of the statistically optimal cutoff.

### Statistical analyses

All statistical analyses were performed using SPSS software (version 23 for Windows; IBM Corp., Armonk, NY, USA). Continuous variables are expressed as medians (interquartile range), and categorical variables are expressed as numbers and percentages. Significant differences in categorical variables between the two groups were analyzed using the chi-square test and Fisher’s exact test. Significant differences in continuous variables between the two groups were compared using the Mann–Whitney U test. The correlation coefficient between the two continuous variables was obtained using the Pearson correlation analysis. The cumulative and ESRD-free survival rates of patients were analyzed using Kaplan–Meier survival analysis. The multivariable Cox hazard model using variables with *P* values < 0.05 in the univariable Cox hazard model was conducted to appropriately obtain the HRs during the follow-up period. *P* values < 0.05 were considered statistically significant.

## Results

### Patient characteristics

The median age of the patients at diagnosis was 61.0 years. Of the 180 patients, 60 (33.3%) patients were men and 122 (67.8%) patients were classified as having MPA. ANCAs were detected in 156 patients (86.7%), of which 5 patients had both types of ANCA. The median values of BVAS, FFS, ESR, and CRP were 12.0, 1.0, 62.0 mm/h, and 14.2 mg/L, respectively. The median calculated FIB-5 was 3.8. The most common comorbidity was hypertension (40.0%), followed by chronic kidney disease (31.7%). During the follow-up, 25 (13.9%) patients died and 37 (20.6%) patients required renal replacement therapy. Glucocorticoids were administered in 92.8% patients. The most frequently administered immunosuppressive drug was azathioprine (53.3%), followed by cyclophosphamide (50.0%) (Table 1).

**Table 1:** Characteristics of 180 patients with MPA and GPA

Variables	Values
At the time of diagnosis	
*Demographic data*	
Age (years)	61.0 (19.0)
Male gender (N, (%))	60 (33.3)
*AAV Subtypes (N, (%))*	
MPA	122 (67.8)
GPA	58 (32.2)
*ANCA positivity (N, (%))*	
MPO-ANCA (or P-ANCA) positivity	129 (71.7)
PR3-ANCA (or C-ANCA) positivity	32 (17.8)
Both ANCA positivity	5 (2.8)
ANCA negativity	24 (13.3)
*AAV-specific indices*	
BVAS	12.0 (11.0)
FFS	1.0 (1.0)
*Acute phase reactants*	
ESR (mm/hr)	62.0 (71.0)
CRP (mg/L)	14.2 (74.9)
*FIB-5 related laboratory results*	
Platelet count (× 10^9^/L)	290.0 (170.0)
Serum albumin (g/L)	36.0 (12.0)
ALP (IU/L)	69.0 (38.0)
AST (IU/L)	18.0 (8.0)
ALT (IU/L)	15.0 (14.0)
FIB-5^*^	3.88 (9.54)
*Comorbidities at diagnosis (N, (%))*	
Chronic kidney disease (stage 3–5)	57 (31.7)
Diabetes mellitus	45 (25.0)
Hypertension	72 (40.0)
Dyslipidaemia	29 (16.1)
Interstitial lung disease	51 (28.3)

*During follow-up*	
*Poor outcomes*	
All-cause mortality (N, (%))	25 (13.9)
Follow-up duration based on mortality (months)	35.7 (62.2)
ESRD (N, (%))	37 (20.6)
Follow-up duration based on ESRD (months)	24.7 (58.2)
*Medications (N, (%))*	
Glucocorticoid	167 (92.8)
Cyclophosphamide	90 (50.0)
Rituximab	32 (17.8)
Azathioprine	96 (53.3)
Mycophenolate mofetil	24 (13.3)
Methotrexate	22 (12.2)

Values are expressed as a median (interquartile range, IQR) or N (%)

MPA: microscopic polyangiitis; GPA: granulomatosis with polyangiitis; AAV: ANCA-associated vasculitis; ANCA: antineutrophil cytoplasmic antibody; MPO: myeloperoxidase; P: perinuclear; PR3: proteinase 3; C: cytoplasmic; BVAS: Birmingham vasculitis activity score; FFS: five-factor score; ESR: erythrocyte sedimentation rate; CRP: C-reactive protein; FIB-5: fibrosis-5; ALP: alkaline phosphatase; AST: aspartate aminotransferase; ALT: alanine aminotransferase; ESRD: end-stage renal disease

### Correlation of FIB-5 and AAV-specific indices at diagnosis

We investigated whether measurement of FIB-5 at diagnosis could reflect the cross-sectional AAV-specific indices, BVAS and FFS. Neither BVAS (*r* = -0.001, *P* = 0.180) nor FFS (*r* = −0.119, *P* = 0.114) at diagnosis was significantly correlated with FIB-5.

### Optimal cutoff value of FIB-5 at diagnosis for the determination of poor outcomes during the follow-up

Using the ROC curve, we extrapolated the optimal cutoff value of FIB-5 at diagnosis for each poor outcome during the follow-up; however, we could not find any statistically significant value. Therefore, the optimal cutoff value of FIB-5 was set as the upper limit of either the lowest tertile (FIB-5 < 0.82 at diagnosis) or the lowest quartile (FIB-5 < -0.42 at diagnosis). The two cutoff values (0.82 and −0.42) were applied to the cumulative survival analysis in this study and the statistically significant cutoff values were selected for the determination of the poor outcomes of AAV.

### Cumulative survival rates of all-cause mortality and ESRD based on the cutoff values of FIB-5 at diagnosis

**Fig. 1:**
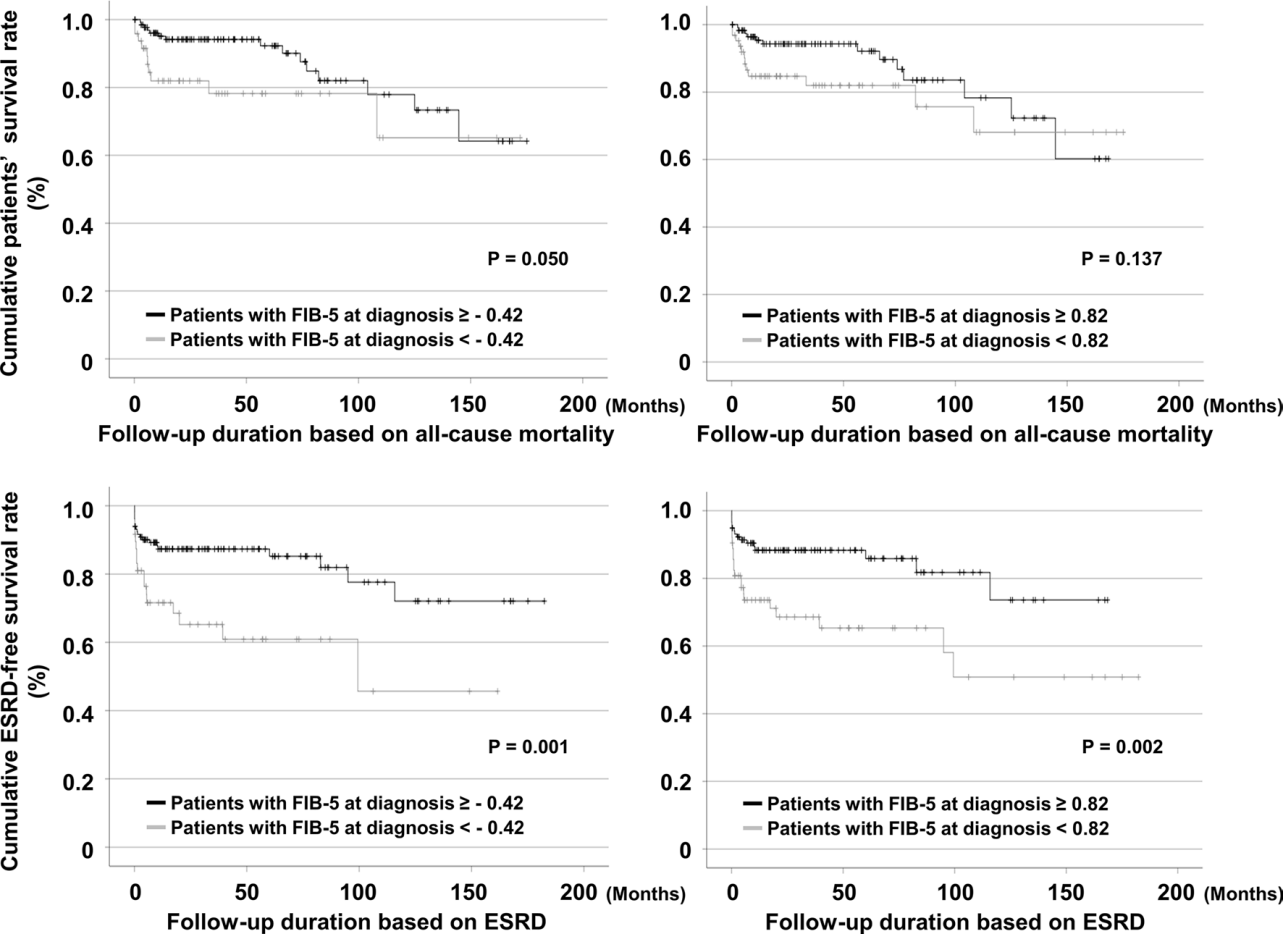
Cumulative and ESRD-free survival rates of patients based on the cutoff values of FIB-5 at diagnosis. Patients with MPA and GPA with FIB-5 < −0.42 at diagnosis had a lower survival rate than those with values ≥ −0.42; however, the difference was not significant. Patients with MPA and GPA with FIB-5 < 0.82 and FIB-5 < −0.42 at diagnosis had lower ESRD-free survival rates than those with FIB-5 ≥ 0.82 and FIB-5 ≥ −0.42. ESRD: end-stage renal disease; FIB-5: Fibrosis-5; MPA: microscopic polyangiitis; GPA: granulomatosis with polyangiitis

In terms of ESRD occurrence, both FIB-5 < −0.42 (*P* = 0.001) and FIB-5 < 0.82 (*P* = 0.002) at diagnosis could significantly predict ESRD during follow-up in MPA and GPA patients (Fig. 1). In terms of all-cause mortality, FIB-5 < −0.42 (*P* = 0.050) at diagnosis tended to presuppose all-cause mortality; however, it did not reach a statistical significance. Whereas, FIB-5 < 0.82 (*P* = 0.137) at diagnosis was not useful to predict all-cause mortality in MPA and GPA patients (Fig. 1). Therefore, FIB-5 < −0.42 at diagnosis was chosen as the cutoff in this study, because of the expectation that the lowest value of FIB-5 could be useful in predicting both all-cause mortality and ESRD in the future study with a larger number of patients.

### Cox hazards model analyses of variables at diagnosis for the occurrence of ESRD during the follow-up

In the univariable Cox hazards model analysis, BVAS, FFS, serum albumin levels, ALT levels, hypertension, and FIB-5 < 0.82 and < −0.42 at diagnosis were significantly associated with the occurrence of ESRD during the follow-up period. In the multivariable analysis with FIB-5 < 0.82 at diagnosis, FFS (HR 1.554, 95% confidence interval [CI] 1.080, 2.236), and FIB-5 values < 0.82 (HR 2.096, 95% CI 1.081, 4.067) independently predicted the occurrence of ESRD. Similarly, in the multivariable analysis with FIB-5 value < -0.42 at diagnosis, FFS (HR 1.534, 95% CI 1.069, 2.201) and FIB-5 < −0.42 (HR 2.073, 95% CI 1.061, 4.050) independently predicted the occurrence of ESRD (Table 2).

**Table 2:** Cox hazards model analysis of variables at diagnosis for presupposing ESRD during follow-up in patients with MPA and GPA

Variables	Univariable			Multivariable (FIB-5 < 0.082)			Multivariable (FIB-5 < −0.042)		
	HR	95% CI	P value	HR	95% CI	P value	HR	95% CI	P value
Age	1.011	0.987, 1.036	0.359						
Male gender	1.251	0.605, 2.587	0.546						
ANCA positivity	3.690	0.882, 15.442	0.074						
BVAS	1.080	1.034, 1.129	0.001	1.041	0.991, 1.093	0.108	1.042	0.991, 1.095	0.105
FFS	2.009	1.438, 2.808	< 0.001	1.554	1.080, 2.236	0.018	1.534	1.069, 2.201	0.020
ESR	1.005	0.997, 1.013	0.227						
CRP	1.004	0.999, 1.009	0.155						
Platelet count	0.999	0.997, 1.001	0.433						
Serum albumin	0.589	0.390, 0.892	0.012	0.661	0.421, 1.037	0.072	0.653	0.415, 1.027	0.065
ALP	1.000	0.996, 1.003	0.935						
AST	0.960	0.916, 1.005	0.083						
ALT	0.933	0.890, 0.978	0.004	0.965	0.925, 1.006	0.097	0.966	0.926, 1.007	0.105
Chronic kidney disease (stage 3–5)	1.274	0.655, 2.478	0.476						
Diabetes mellitus	1.123	0.543, 2.322	0.755						
Hypertension	2.367	1.227, 4.565	0.010	1.802	0.915, 3.549	0.089	1.749	0.887, 3.449	0.106
Dyslipidaemia	1.440	0.658, 3.150	0.362						
FIB-5 < 0.082	2.708	1.411, 5.195	0.003	2.096	1.081, 4.067	0.029			
FIB-5 < -0.042	2.785	1.454, 5.334	0.002				2.073	1.061, 4.050	0.033

ESRD: end-stage renal disease; MPA: microscopic polyangiitis; GPA: granulomatosis with polyangiitis; FIB-5: fibrosis-5; HR: hazard ratio; CI: confidence interval; ANCA: antineutrophil cytoplasmic antibody; BVAS: Birmingham vasculitis activity score; FFS: five-factor score; ESR: erythrocyte sedimentation rate; CRP: C-reactive protein; ALP: alkaline phosphatase; AST: aspartate aminotransferase; ALT: alanine aminotransferase

## Discussion

In this study, we investigated whether measurement of FIB-5 at diagnosis could predict all-cause mortality and the occurrence of ESRD during the follow-up period in patients with MPA and GPA. We demonstrated, for the first time, that FIB-5 was an independent predictor of the occurrence of ESRD, but not all-cause mortality. However, we could not prove that measurement of FIB-5 at diagnosis reflected the cross-sectional indices BVAS and FFS.

We compared FIB-5 values at diagnosis based on the presence of each item of the BVAS, rather than considering the total score. However, we did not find any differences in the FIB-5 values based on the absence or presence of each item. Differences in FIB-5 values at diagnosis between patients with renal manifestations and those without were expected; however, the difference was not statistically significant (2.63 vs. 4.85, *P* = 0.173). In addition, we also compared FIB-5 values at diagnosis based on the presence of each comorbidity. Patients with chronic kidney disease had lower FIB-5 values at diagnosis than those without (3.33 vs. 3.97, *P* = 0.071); however, the difference was not statistically significant. In contrast, patients with dyslipidaemia exhibited significantly lower FIB-5 values at diagnosis than those without (0.97 vs. 3.97, *P* = 0.031) (Supplementary Table 1).

Chronic kidney disease is a major risk factor for the development of ESRD [3, 4]. Therefore, elucidation of the association between FIB-5 and chronic kidney disease could aid in understanding the ability of FIB-5 in prediction the development of ESRD. Based on this concept, we assessed the relative risk of FIB-5 < −0.42 and FIB-5 < 0.82 at diagnosis on concurrent chronic kidney disease. Chronic kidney disease was identified more frequently in patients when the optimal cutoff value of FIB-5 at diagnosis was set as −0.42 compared to when the optimal cutoff value of FIB-5 at diagnosis was set as ≥ −0.42 (43.8% vs. 27.3%, *P* = 0.036). Furthermore, patients with FIB-5 < −0.42 at diagnosis had a significantly higher risk for concurrent chronic kidney disease than those with FIB-5 ≥ −0.42 (relative risk [RR] 2.074, 95% CI 1.043, 4.123). Furthermore, concurrent chronic kidney disease was not different between the two groups when the cutoff value of FIB-5 was set as 0.82 (Supplementary Fig. 1).

In addition to identification of concurrent chronic kidney disease during diagnosis of MPA and GPA, we also investigated the change in FIB-5 values based on the degree of renal function decline. Blood urea nitrogen (*r* = −0.326, *P* < 0.001) and serum creatinine (*r* = −0.24, *P* < 0.001) levels significantly and negatively correlated with FIB-5 values at diagnosis (Supplementary Fig. 2). Furthermore, we analyzed the association between FIB-5 values at diagnosis and other risks of ESRD in patients with MPA and GPA. However, we did not observe any association between FIB-5 and MPO-ANCA, ANCA positivity, and hypertension at diagnosis [3]. Based on these results, we concluded that FIB-5 might be associated with concurrent chronic kidney disease or the extent of reduced renal function at diagnosis, which are well-known risk factors for occurrence of ESRD in patients with MPA and GPA. It may be considered that measurement of FIB-5 at diagnosis could predict ESRD during follow-up in patients with MPA and GPA.

We have two reasons why we did include chronic kidney disease (stage 3–5) rather than serum creatinine in the multivariable Cox hazards model analysis for ESRD occurrence. One reason is that chronic kidney disease (stage 3–5) without renal replacement therapy is classified based on estimated glomerular filtration rate which is calculated by several parameters, particularly serum creatinine. Therefore, in order to avoid overlapping application, chronic kidney disease rather than serum creatinine was chosen. The other reason is that chronic kidney disease (stage 3–5) is a more stable parameter than serum creatinine. For, serum creatinine can be altered by diverse medical conditions such as excess of exercise and nutritional status, whereas a diagnosis of chronic kidney disease is usually made through tests at several visits more than once.

To date, one study has reported on metabolic syndrome in AAV on the basis of a previous study [17]; however, there are no studies on the direct association between FIB-5 and dyslipidaemia at diagnosis. Considering the number of studies on the association between FIB-5 and chronic kidney disease at diagnosis and occurrence of ESRD during follow-up, it may be speculated that FIB-5 and dyslipidaemia might be associated with serious thrombotic events during follow-up of patients with MPA and GPA. Based on this concept, we compared the cumulative cerebrovascular accident (CVA)- and cardiovascular disease (CVD)-free survival rates between patients with FIB-5 < −0.42 and those with FIB-5 ≥ −0.42 at diagnosis. However, there were no differences in the cumulative CVA- and CVD-free survival rates between the two groups (*P* = 0.174 and *P* = 0.326) (Supplementary Fig. 3).

The extended systemic inflammation may initiate and aggravate the fibrotic process. Particularly, endothelial damage may accelerate both innate and adaptive immune activation by various inflammatory cytokines including interleukin (IL)-1, IL-6 and tumor necrosis factor (TNF)-α. Subsequently, activated innate immune cells including macrophages and polymorphonuclear leukocytes, and polarized helper T cells toward inflammation and activated autoreactive B cells may drive fibroblast activation, resulting in a formation of a vicious cycle of fibrosis in any organs [18, 19]. On the basis of this immunological background, we suggested three hypotheses supporting our assumption that FIB-5 can predict ESRD in patients with MPA and GPA in the Introduction section. First, myofibroblast also may share the interaction with not only liver and kidney-specific cells, but also general cells associated with fibrosis [10]. Second, subclinical liver disease may occur in response to systemic inflammation and subsequently may affect the serum levels of AST, ALT, and alkaline phosphatase [11–13]. Third, FIB-5 includes indicators of systemic inflammation [14].

A previous study proved that FIB-4 at diagnosis was an independent indicator for predicting all-cause mortality in patients with MPA and GPA [7]. Meanwhile, this study demonstrated that FIB-5 at diagnosis could not predict all-cause mortality in those patients (Supplementary Table 2). What made these differences between FIB-4 and FIB-5? The most critical differences in parameters between equations of FIB-4 and FIB-5 are the age of FIB-4 and serum albumin of FIB-5. Age is a well-known conventional risk factor of all-cause mortality, whereas, serum albumin is closely associated with renal impairment in patients with MPA and GPA [20, 21]. For these reasons, FIB-4 may anticipate all-cause mortality better than FIB-5, conversely, FIB-5 may predict ESRD occurrence better than FIB-4 in patients with MPA and GPA during follow-up.

The merit of our study is that the clinical implication of FIB-5 measurement at diagnosis in patients with MPA and GPA has been investigated for the first time. However, this study has several limitations. Due to the limitations of the retrospective study design, accurate information could not be obtained on medications other than those administered for the comorbidities or immunosuppressive agents at the time of diagnosis. Despite including the largest number of patients in South Korea, this was a single institutional study; therefore, the sample size could be considered insufficient to represent all South Korean patients with MPA and GPA. Although FIB-5 is an index that reflects liver fibrosis, this study did not include objective data for assessment of liver fibrosis through transient elastography or ultrasonography. A prospective multi-center study with a larger number of patients together with data on liver fibrosis would provide more reliable information regarding clinical usefulness of FIB-5 in patients with MPA and GPA.

In conclusion, measurement of FIB-5 at diagnosis could not reflect the cross-sectional indices BVAS and FFS; however, FIB-5 < 0.82 and FIB-5 < −0.42 at diagnosis could predict the occurrence of ESRD during follow-up in patients with MPA and GPA.

## Supplementary information

Below is the link to the electronic supplementary material.

Supplementary file1 (TIF 567 KB)

Supplementary file2 (TIF 1512 KB)

Supplementary file3 (TIF 914 KB)

Supplementary file4 (DOCX 13 KB)

Supplementary file5 (DOCX 18 KB)

## Data Availability

The data analyzed are available from the corresponding author on reasonable request.
