# Supplementary figures and images for: Fibrosis-5 predicts end-stage renal disease in patients with microscopic polyangiitis and granulomatosis with polyangiitis without substantial liver diseases
Source: Clin Exp Med. 2021 Feb 20;21(3):399–406. doi: 10.1007/s10238-021-00691-2 (PMC8266773; doi:10.1007/s10238-021-00691-2)

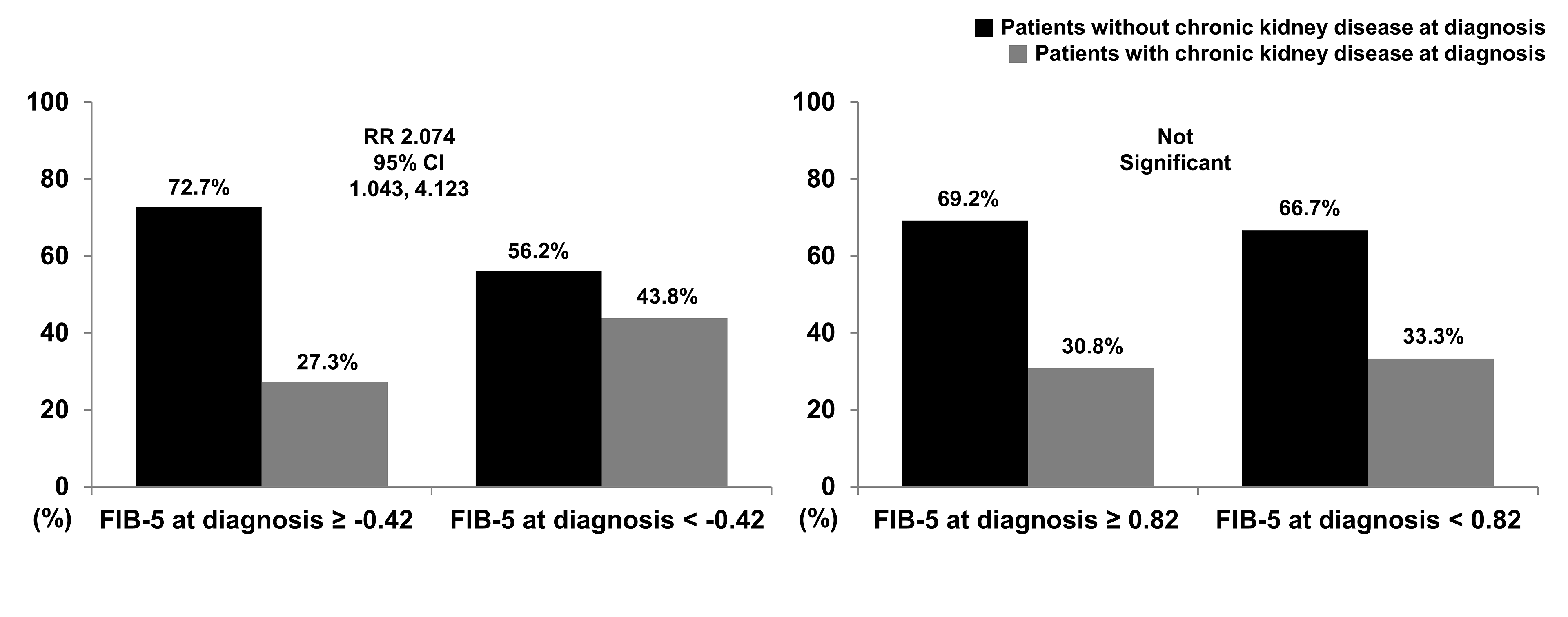

Supplement: Supplementary file 1 — Supplementary file1 (TIF 567 KB) [file 10238_2021_691_MOESM1_ESM.tif]

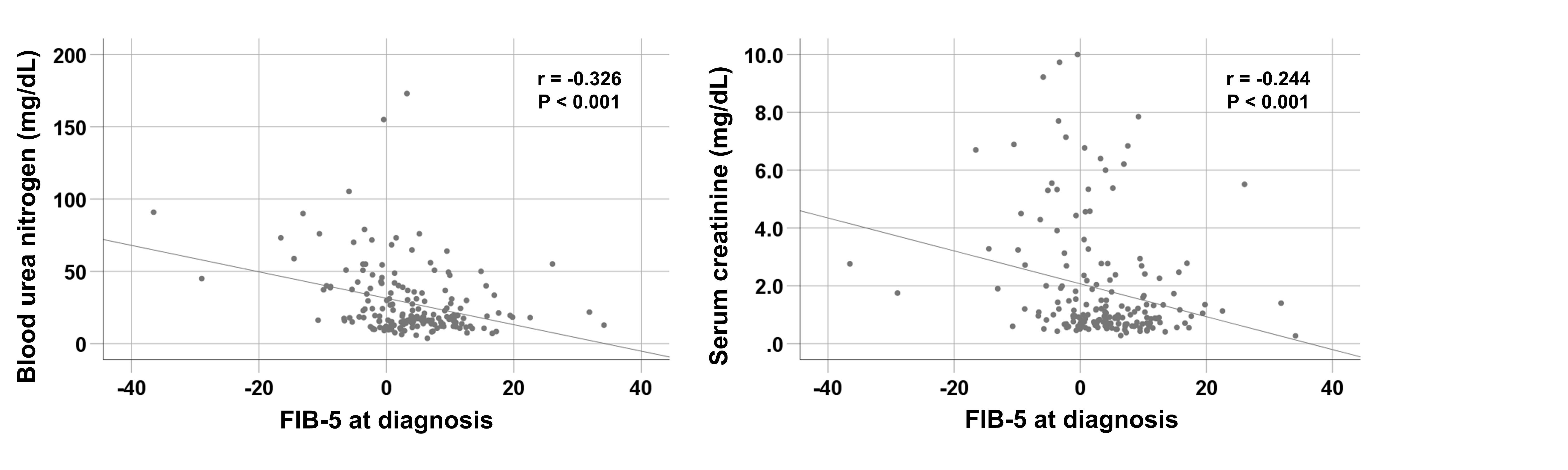

Supplement: Supplementary file 2 — Supplementary file2 (TIF 1512 KB) [file 10238_2021_691_MOESM2_ESM.tif]

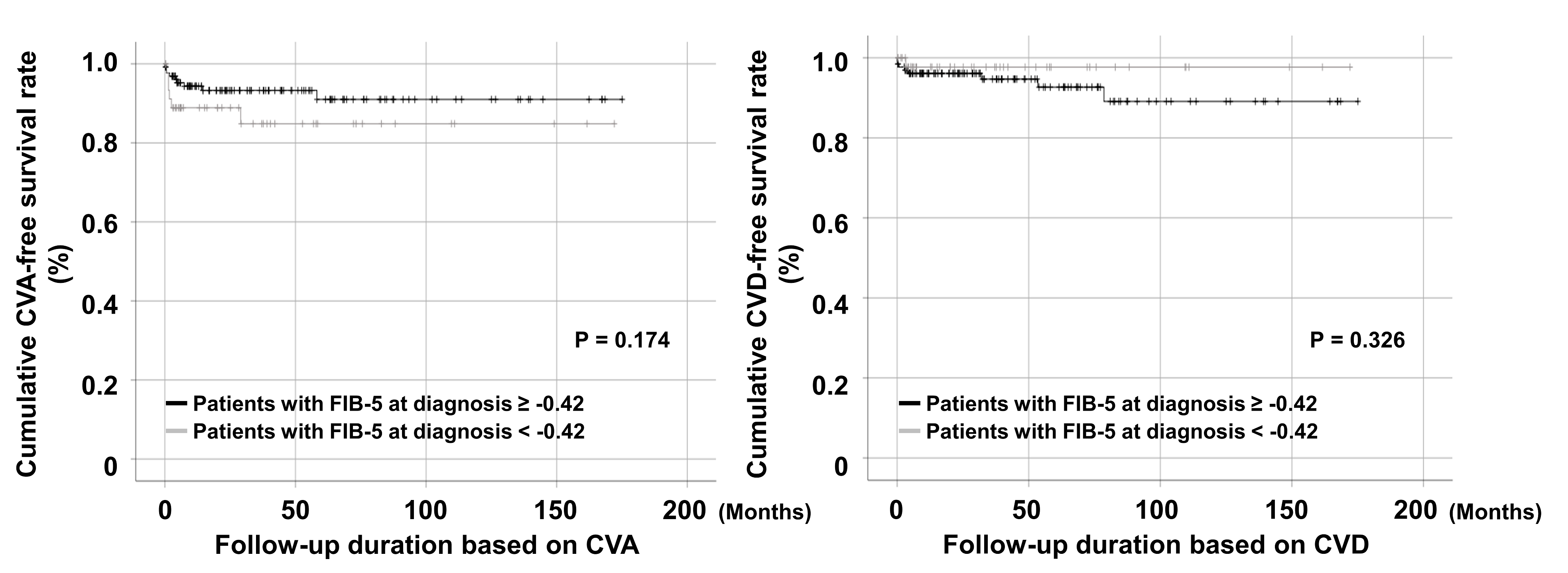

Supplement: Supplementary file 3 — Supplementary file3 (TIF 914 KB) [file 10238_2021_691_MOESM3_ESM.tif]
