# Supplementary material for: Fibrosis-5 predicts end-stage renal disease in patients with microscopic polyangiitis and granulomatosis with polyangiitis without substantial liver diseases
Source: Clin Exp Med. 2021 Feb 20;21(3):399–406. doi: 10.1007/s10238-021-00691-2 (PMC8266773; doi:10.1007/s10238-021-00691-2)
Supplement: Supplementary file 4 — Supplementary file4 (DOCX 13 KB) [file 10238_2021_691_MOESM4_ESM.docx]

**Supplementary Table1. Comparison of FIB-5 between absence and presence of either item of BVAS or comorbidities**

|  | **Absence** | **Presence** | **P-value** |
| --- | --- | --- | --- |
| ***Items of BVAS*** |  |  |  |
| General | 4.10 (8.42) | 3.34 (11.68) | 0.314 |
| Cutaneous | 3.94 (10.33) | 3.23 (7.54) | 0.834 |
| Mucocutaneous/Eyes | 3.82 (9.85) | 3.90 (7.57) | 0.905 |
| ENT | 2.60 (7.86) | 4.76 (10.69) | 0.193 |
| Pulmonary | 3.63 (7.62) | 3.87 (10.66) | 0.812 |
| Cardiovascular | 3.51 (9.43) | 4.30 (9.91) | 0.769 |
| Gastrointestinal | 3.90 (9.76) | -0.28 (8.93) | 0.197 |
| Renal | 4.85 (8.87) | 2.63 (9.97) | 0.173 |
| Nervous | 3.05 (9.63) | 4.06 (11.65) | 0.382 |
| ***Comorbidities*** |  |  |  |
| Chronic kidney disease (stage 3-5) | 3.97 (9.27) | 3.33 (10.34) | 0.071 |
| Diabetes mellitus | 3.92 (9.90) | 3.82 (8.19) | 0.826 |
| Hypertension | 3.51 (9.15) | 4.07 (11.11) | 0.684 |
| Dyslipidaemia | 3.97 (9.52) | 0.97 (10.32) | 0.031 |
| Interstitial lung disease | 3.36 (10.00) | 4.08 (8.55) | 0.757 |
