# Supplementary material for: Fibrosis-5 predicts end-stage renal disease in patients with microscopic polyangiitis and granulomatosis with polyangiitis without substantial liver diseases
Source: Clin Exp Med. 2021 Feb 20;21(3):399–406. doi: 10.1007/s10238-021-00691-2 (PMC8266773; doi:10.1007/s10238-021-00691-2)
Supplement: Supplementary file 5 — Supplementary file5 (DOCX 18 KB) [file 10238_2021_691_MOESM5_ESM.docx]

**Supplementary Table 2 Cox hazards model analysis of variables at diagnosis for presupposing all-cause mortality during follow-up in patients with MPA and GPA**

| **Variables** | **Univariable** | | |
| --- | --- | --- | --- |
|  | **HR** | **95% CI** | **P value** |
| Age | 1.049 | 1.031, 1.087 | 0.008 |
| Male gender | 1.913 | 0.862, 4.247 | 0.111 |
| ANCA positivity | 1.547 | 0.507, 4.716 | 0.443 |
| BVAS | 1.088 | 1.036, 1.144 | 0.001 |
| FFS | 2.376 | 1.565, 3.609 | <0.001 |
| ESR | 1.003 | 0.993, 1.013 | 0.560 |
| CRP | 1.007 | 1.001, 1.013 | 0.021 |
| Platelet count | 1.000 | 0.998, 1.003 | 0.093 |
| Serum albumin | 0.357 | 0.208, 0.612 | <0.001 |
| ALP | 1.003 | 1.001, 1.006 | 0.014 |
| AST | 1.013 | 1.004, 1.022 | 0.006 |
| ALT | 1.004 | 0.997, 1.012 | 0.212 |
| Chronic kidney disease (stage 3-5) | 2.001 | 0.911, 4.396 | 0.084 |
| Diabetes mellitus | 0.886 | 0.353, 2.225 | 0.797 |
| Hypertension | 1.215 | 0.551, 2.681 | 0.630 |
| Dyslipidaemia | 2.045 | 0.852, 4.911 | 0.109 |
| FIB-5 < 0.082 | 1.800 | 0.820, 3.952 | 0.143 |
| FIB-5 < -0.042 | 2.189 | 0.981, 4.882 | 0.056 |

MPA: microscopic polyangiitis; GPA: granulomatosis with polyangiitis; HR: hazard ratio; CI: confidence interval; ANCA: antineutrophil cytoplasmic antibody; BVAS: Birmingham vasculitis activity score; FFS: five factor score; ESR: erythrocyte sedimentation rate; CRP: C-reactive protein; ALP: alkaline phosphatase; AST: aspartate aminotransferase; ALT: alanine aminotransferase; FIB-5: fibrosis-5.
